# CCAAT/enhancer-binding protein delta regulates the stemness of glioma stem-like cells through activating PDGFA expression upon inflammatory stimulation

**DOI:** 10.1186/s12974-019-1535-z

**Published:** 2019-07-12

**Authors:** Shao-Ming Wang, Hong-Yi Lin, Yen-Lin Chen, Tsung-I Hsu, Jian-Ying Chuang, Tzu-Jen Kao, Chiung-Yuan Ko

**Affiliations:** 1 Graduate Institute of Neural Regenerative Medicine, College of Medical Science and Technology, Taipei Medical University, Taipei, Taiwan; 2 Ph.D. Program for Neural Regenerative Medicine, College of Medical Science and Technology, Taipei Medical University and National Health Research Institutes, Taipei, Taiwan; 3 Department of Pathology, Cardinal Tien Hospital, School of Medicine College of Medicine, Fu Jen Catholic University, New Taipei City, Taiwan; 4 Research Center of Neuroscience, Taipei Medical University, Taipei, Taiwan; 5 TMU Research Center of Cancer Translational Medicine, Taipei, Taiwan

**Keywords:** Glioblastoma multiforme, Glioma stem-like cell, CEBPD, PDGFA, Inflammation, Interleukin-1β

## Abstract

**Background:**

The small population of glioma stem-like cells (GSCs) contributes to tumor initiation, malignancy, and recurrence in glioblastoma. However, the maintenance of GSC properties in the tumor microenvironment remains unclear. In glioma, non-neoplastic cells create an inflammatory environment and subsequently mediate tumor progression and maintenance. Transcriptional factor CCAAT/enhancer-binding protein delta (CEBPD) is suggested to regulate various genes responsive to inflammatory cytokines, thus prompting us to investigate its role in regulating GSCs stemness after inflammatory stimulation.

**Methods:**

Stemness properties were analyzed by using spheroid formation. Oncomine and TCGA bioinformatic databases were used to analyze gene expression. Western blotting, quantitative real-time polymerase chain reaction, luciferase reporter assay, and chromatin immunoprecipitation assay were used to analyze proteins and gene transcript levels. The glioma tissue microarrays were used for CEBPD and PDGFA expression by immunohistochemistry staining.

**Results:**

We first found that IL-1β promotes glioma spheroid formation and is associated with elevated CEBPD expression. Using microarray analysis, platelet-derived growth factor subunit A (PDGFA) was confirmed as a CEBPD-regulated gene that mediates IL-1β-enhanced GSCs self-renewal. Further analysis of the genomic database and tissue array revealed that the expression levels between CEBPD and PDGFA were coincident in glioma patient samples.

**Conclusion:**

This is the first report showing the activation of PDGFA expression by CEBPD through IL-1β treatment and a novel CEBPD function in maintaining the self-renewal feature of GSCs.

**Electronic supplementary material:**

The online version of this article (10.1186/s12974-019-1535-z) contains supplementary material, which is available to authorized users.

## Background

As one of the most common and lethal brain tumor types, glioblastoma multiforme (GBM) contains neoplastic and non-neoplastic cells, including glioma stem-like cells (GSCs), microglia, astrocytes, and endothelial cells [1–3]. Glioma stem-like cells contribute to the tumor-initiating activity and tumor recurrence through the stem cell-like characteristics, including their self-renewal ability and the expression of neural stem cell markers [4–6]. Several studies report that the microenvironment serves as one of the key factors to promote cancer progression and malignancy in glioma [1, 3, 7]. In brain tumors, the majority of stromal cells are brain-intrinsic microglia/macrophages, which support tumor cell growth and invasion [3]. Moreover, glioma cells can release several chemotactic factors, which attract microglia/macrophages to the tumor bulk, and these microglia/macrophages can further produce several inflammatory factors to promote glioma cell invasion [3, 8]. However, whether these inflammatory cytokines can promote GSCs feature remains unclear.

CCAAT/enhancer-binding protein delta belongs to the CCAAT/enhancer-binding protein (C/EBP) family. The expression of CEBPD protein is regulated by a variety of extracellular stimuli, such as IL-1β and tumor necrosis factor (TNF)-α [9, 10]. The biological functions of CEBPD include cell migration, anti-apoptosis, ROS formation, and differentiation [10–13]. In previous cancer studies, CEBPD has been suggested to induce cell growth arrest and apoptosis in hepatocellular carcinoma and prostate cancer and thus function as a tumor suppressor [14, 15]. On the other hand, recent studies imply that CEBPD plays a pro-tumorigenic role in drug resistance and cell invasion in bladder cancer [16, 17]. According to GEO profile data (GSE2223, GSE4290, and GSE4536) [18–20], CEBPD is expressed in GBM at a higher level when comparing with that in normal brain tissues. However, the functions of CEBPD in glioma and inflammation-induced GSC stemness remain to be clarified.

The activation of PDGF/PDGFR signaling can trigger cancer progression and malignancy [2, 21]. PDGFs are secretory factors, and four members have been identified, including PDGFA, B, C, and D [22]. Among them, PDGFA is found in 70% of gliomas and is highly expressed in GBM [2, 22]. Previous studies indicate that PDGFA can promote glioma proliferation, invasion, and self-renewal [2, 23], thus suggesting that PDGFA regulates GSC stemness and promotes glioma progression. However, whether GSCs express PDGFA in an inflammatory environment is still unknown, and the molecular mechanism driving the change of PDGFA expression in GSCs remains unclear.

In this study, we propose that CEBPD plays a regulatory role in inflammatory responses and contributes to GSC stemness in GBM. To test this hypothesis, we investigate the function of CEBPD in cultured glioma spheroids in vitro. We first show that IL-1β is highly expressed in GBM, promotes glioma spheroid formation, and is associated with the elevated CEBPD expression. Focusing on GSC stemness, we then show that CEBPD plays a functional role of inducing the stem-like feature in the inflammatory environment. We further demonstrate that PDGFA is responsive to CEBPD induction in glioma spheroid cells, and CEBPD regulates *PDGFA* transcription by binding to the *PDGFA* promoter region. Combined, our findings indicate that CEBPD is required for IL-1β-driven self-renewal of GSCs, and the PDGFA expression regulated by CEBPD is a key mechanism for GSCs self-renewal in the inflammatory environment.

## Methods

### Cell culture and transfection

The human U373MG cell line (human glioblastoma astrocytoma) and human T98G cell line (human glioblastoma multiforme) were purchased from the American Type Culture Collection (ATCC) and were cultured in Dulbecco’s modified Eagle medium (DMEM, Thermo Fisher Scientific, MA, USA) containing 10% fetal bovine serum (FBS, Thermo Fisher Scientific, MA, USA), 100 units/mL penicillin, and 100 μg/mL streptomycin (Thermo Fisher Scientific, MA, USA). We confirmed the authentication of all cell lines by short tandem repeat (STR) analyses of cell DNA alleles. Cells were incubated in a humidified incubator at 37 °C with 5% CO_2_. The siRNA knockdown for siControl (4390843, Ambion, CA, USA) and siCEBPD (si2895: 5′-UUCUCUCGCAGUUUAGUGGTG-3′; si2896: 5′-AUUGCUGUUGAAGAGGUCGGC-3′) was purchased from Thermo (Ambion, CA, USA). The cells were transfected with siRNA using specific siRNA Lipofectamine reagent (RNAiMAX reagent, Thermo Fisher Scientific, MA, USA).

### Isolation and culture of primary glioblastoma cells

The use of human specimens was approved by the Institute Review Board (IRB)/Ethics Committee from the office of human research in Taipei Medical University (Taipei, Taiwan). The consent of the patient was obtained and approved by Taipei Medical University IRB protocols (N201006011). PT#3 glioblastoma cells were isolated from the glioblastoma tissue of a male patient. The patient was treated and taken care in the Taipei Medical University Hospital (Taipei, Taiwan). The freshly resected tissues were digested by 0.05% collagenase type IV (Sigma-Aldrich, St. Louis, MO, USA) and 5 units/mL DNase I (Sigma-Aldrich, St. Louis, MO, USA) at 37 °C for 2 h. After removing undigested tissues by centrifugation, the supernatant was mixed with DMEM supplemented with 10% fetal bovine serum (FBS, Thermo Fisher Scientific, MA, USA) and transferred to 6-well culture plates. Cells were then maintained in DMEM-10% FBS.

### GEO database

The Oncomine database was used to assess the gene expression in normal and GBM tissues. The accession numbers of GEO datasets used in this study are Bredel dataset (GSE2223), Sun dataset (GSE4290), and Lee dataset (GSE4536).

### Tumor spheroid formation assay

In tumor spheroid cultures, cells were seeded on poly (2-hydroxyethyl methacrylate) (Santa Cruz Biotechnology, Inc., Dallas, TX, USA)-coated 6-cm dish and cultured in DMEM/F12 medium (Invitrogen, Carlsbad, CA, USA) containing N2 supplement (Invitrogen, Carlsbad, CA, USA), 20 ng/mL EGF (Invitrogen, Carlsbad, CA, USA), and 20 ng/mL FGF (Invitrogen, Carlsbad, CA, USA).

### Real-time quantitative reverse transcriptase PCR

Total RNA was extracted using the Direct-zol™ RNA MiniPrep kit (Zymo Research, CA, USA) from TRIsure RNA extraction reagent. The synthesis of complementary DNA (cDNA) was completed with reverse transcription (RT) reactions using the PrimeScript™ RT reagent Kit (TAKARA, JPN). Real-time PCR was conducted using iTaq Universal SYBR Green Supermix. PCR was conducted using StepOne Plus™ real-time PCR systems (ABI) with the following pairs of specific primers: human CEBPD (S): 5-′GCCATGTACGACGACGAGAG-3′ and CEBPD (AS): 5-′TGTGATTGCTGTTGAAGAGGTC-3′; PDGFA (S): 5-′AAGTCCAGGTGAGGTTAG-3′ and PDGFA (AS): 5-′TCCTCTTCCCGATAATCC-3′; and GAPDH (S): 5-′GTCTCCTCTGACTTCAACAGCG-3′ and GAPDH (AS): 5-′ACCACCCTGTTGCTGTAGCCAA-3′. All reactions were performed in duplicates with “no reverse transcriptase” as the control, and all data are mean ± SEM of at least three independent biological replicates. The relative expression levels were measured using the relative quantitation (RQ) ΔΔCt method and normalized to the housekeeping gene glyceraldehyde 3-phosphate dehydrogenase (GAPDH).

### Western blot analysis

Cells were harvested and lysed with Pierce RIPA buffer (Thermo, MA, USA). Lysates were resolved on a sodium dodecyl sulfate gel containing 10 or 12% polyacrylamide, and then transferred to a polyvinylidene difluoride (PVDF) nylon membrane and hybridized with primary antibodies against CEBPD (sc-636x, Santa Cruz, CA, USA), PDGFA (sc-9974, Santa Cruz, CA, USA), CD133 (18470-1-AP, Proteintech, HB, CHN), or α-tubulin (T9026, Sigma-Aldrich, MO, USA) at 4 °C overnight. Specific proteins were detected by peroxidase-conjugated secondary antibodies incubated at room temperature for 1 h. Signals were revealed by the enhanced chemiluminescence Western blot system from ChemiDoc Touch™ Imaging System (BIO-RAD, CA, USA).

### Establishment of stably knockdown clones

Virus was produced from Phoenix cells by cotransfection of the various small hairpin RNA expression vectors in combination with pMD2.G and psPAX2. After determining the viral infection efficiency, 10 multiplicity of infection of lentivirus containing shβ-galactosidase (shLacZ, for sham control) or shCEBPD (shC7) were used to infect U373MG or T98G cells for 96 h. Cells were further selected and maintained in 2 μg/mL puromycin (Gibco, MA, USA). In all lentiviral experiments, the medium containing uninfected viruses was removed before further assays were conducted. The small hairpin RNA sequences in lentiviral expression vectors were as follows: shβ-galactosidase: 5′-CCGGTGTTCGCATTATCCGAACCATCTCGAGATGGTTCGGATAATGCGAACATTTTTG-3′ and shCEBPD (shC7): 5′-CCGGGCTGTCGGCTGAGAACGAGAACTCGAGTTCTCGTTCTCAGCCGACAGCTTTTT-3′. The lentiviral knockdown expression vectors were obtained from the National RNAi Core Facility located at the Genomic Research Center of Institute of Molecular Biology, Academia Sinica (Taiwan).

### Microarray analysis

Total RNAs were harvested from U373MG cells transfected with siC or siCEBPD for 24 h. Samples were validated with Agilent Human Whole Genome Oligo 4 × 44 K Microarray (Welgene Biotech. Co., Taipei, Taiwan) following the manufacturer’s protocols. All processes were performed by Welgene Biotech Company (Taipei, Taiwan). Good quality signals were obtained by filtering for scores of *p* value < 0.05 in all replicates, *M* value > 6 in all signals, and > 1.5-fold change.

### Chromatin immunoprecipitation assay

In brief, cells were treated with 1% formaldehyde for 10 min, and the nuclear proteins were extracted. The cross-linked chromatin was then prepared and sonicated to an average length between 200 bp and 1000 bp. The DNA fragments were immunoprecipitated with specific antibodies recognizing CEBPD (sc-636x, Santa Cruz, CA, USA) or control rabbit immunoglobulin G (IgG) (sc-2027, Santa Cruz, CA, USA) at 4 °C for 16 h. After reversal of the crosslinking between proteins and genomic DNA, the precipitated DNA was amplified by PCR with primers related to the specific regions on the genomic loci of target genes. The following primers were used: PDGFA-I forward 5′-GACTCCCCCTCCTTTTATGG-3′ and PDGFA-I reverse 5′-AGTGCCAGCTGCAATCCT-3′, and PDGFA-II forward 5′-GGGGCTTTGATGGATTTAGC-3′ and PDGFA-II reverse 5′-GGCGGGGAGAGGGTTATAG-3′.

### Immunohistochemistry (IHC) analysis

The tissue microarrays were purchased from Biomax (GL481, Rockville, MD, USA). IHC staining was performed using a Ventana BenchMark XT automated stainer (Ventana, Tucson, AZ, USA) and specific primary antibodies against CEBPD (ab65081, Cambridge, MA, USA) and PDGFA (sc-9974, Santa Cruz, CA, USA). The *H*-scores ranging from 0 to 300 were calculated by multiplying the staining intensity by the percentage of each core.

### Statistical analysis

GraphPad Prism 7 software was used for all statistical analysis, and results are shown as means ± SEM. Two-tailed unpaired *t* test and one-way ANOVA followed by Tukey’s multiple comparison test were used. The correlation analysis was determined by the Pearson correlation test. All experiments were repeated three times. Statistically significant differences are indicated by *p < 0.05, **p < 0.01, and ***p < 0.001.

## Results

### IL-1β induces the spheroid formation of glioma stem cells and is associated with the elevated CEBPD expression

**Fig. 1:**
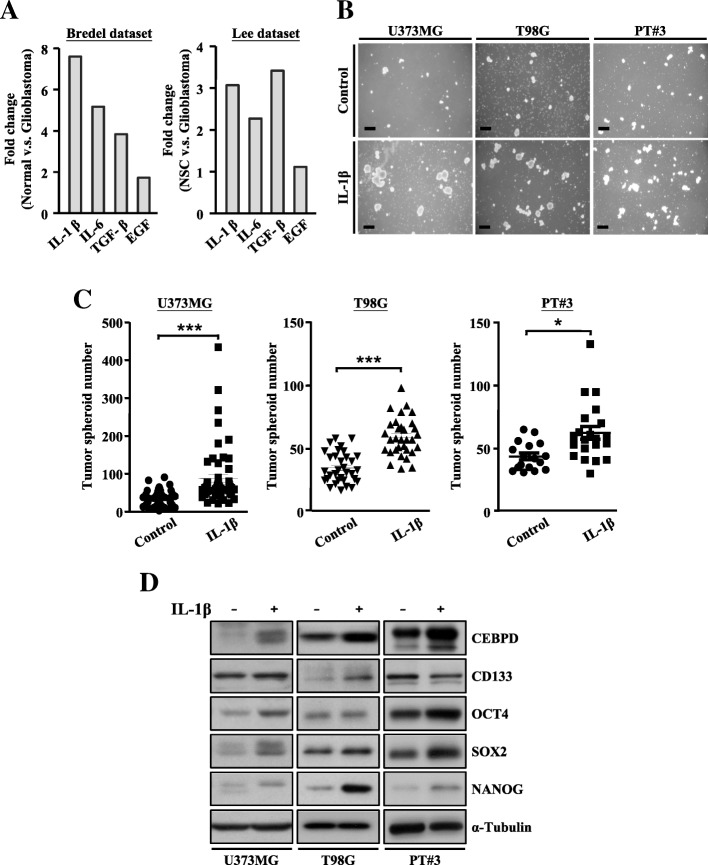
Treatment with IL-1β promotes tumor spheroid formation and is associated with CEBPD expression. **a** Analyses of the GEO database showing higher IL-1β expressions than those of other factors in GBM. **b** IL-1β promoting tumor spheroid formation in U373MG, T98G, and PT#3 cells. Tumor spheroids were incubated in DMEM/F12 medium containing N2 supplement, 20 ng/mL EGF, and 20 ng/mL FGF, and images were collected on day 6. Scale bar, 0.5 mm. **c** Quantitative analysis results of tumor spheroid number in **b** using ImageJ software. **d** Western blot analyses conducted with indicated antibodies using protein lysates from U373MG, T98G, and PT#3 spheroid cells on day 6. Expression of α-tubulin served as the internal control. (Total data were obtained from three independent experiments, and the data shown here were from one representative assay. All data are expressed as mean ± SEM. Differences between groups were determined with the unpaired two-tailed *t* test. *p < 0.05, ***p < 0.001)

In GBM, inflammatory cytokines or growth factors, including IL-1β, IL-6, TGF-β, and EGF, are abundantly produced by microglia/macrophages [3]. From the GEO databases (GSE2223 and GSE4536), we found higher IL-1β expression levels compared with other factors in GBM (Fig. 1a). We thus used IL-1β to mimic the inflammatory environment. To know whether IL-1β stimulation contributes to GSC spheroid formation, glioma cells were subjected to the neurosphere formation assay to test the self-renewal properties of stem cells, upon treatment of IL-1β. Following IL-1β treatment, the numbers of glioma spheroids significantly increased compared to those in untreated controls (Fig. 1b, c). In addition, we found increased expressions of CEBPD gene and stemness-related factors (CD133, SOX2, OCT4, and NANOG) following IL-1β stimulation in U373MG, T98G, and primary patient culture (PT#3)-driving spheroid cells (Fig. 1d). These observations thus indicate that IL-1β promotes GSC spheroids, which may be associated with the upregulated CEBPD expression.

### CEBPD contributes to the glioma stem cell spheroid formation under IL-1β treatment

**Fig. 2:**
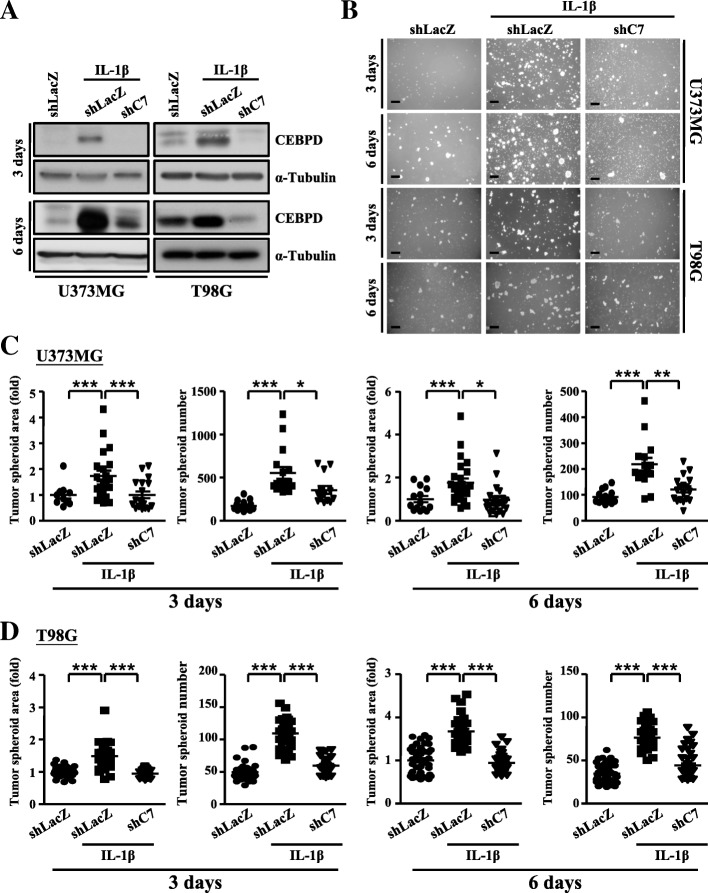
CEBPD contributes to self-renewal in U373MG and T98G spheroid cells under IL-1β treatment. **a** Western blot analyses conducted with indicated antibodies using protein lysates from U373MG or T98G spheroid cells stably expressing shLacZ or shC7 treated with or without 5 ng/mL IL-1β on day 3 and 6. Expression of α-tubulin served as the internal control. **b** Images collected from tumor spheroids of U373MG or T98G cells stably expressing shLacZ or shC7 treated with or without 5 ng/mL IL-1β. Scale bar, 0.5 mm. **c**, **d** Quantitative analysis results of tumor spheroid area and number in **b** using ImageJ software. Cells transduced with shLacZ served as the sham control. (Total data were obtained from three independent experiments, and the data shown here were from one representative assay. All data are expressed as mean ± SEM. Differences among groups were determined with one-way ANOVA followed by Tukey’s multiple comparison test. *p < 0.05, **p < 0.01, ***p < 0.001) shLacZ, shβ-galactosidase; shC7, shCEBPD

Previous studies showed that GSCs were the key drivers of glioma malignancy and progression [1, 2, 4, 5]. According to the GEO database, CEBPD was found expressed in GBM at higher levels compared with those in the normal brain tissue, which is in line with our results showing the association of increased CEBPD expression with GSCs spheroid formation upon IL-1β treatment. To test this idea of CEBPD involvement in the GSCs spheroid formation induced by IL-1β, we generated U373MG and T98G cells that had stable knockdown of CEBPD by lentiviral transduction of shCEBPD. Cells transduced with shLacZ were served as the sham control (Fig. 2a and Additional file 1: Figure S1a). As shown in Fig. 2b–d and Additional file 1: Figures S1 and S2, the knockdown of CEBPD in U373MG and T98G cells significantly reduced the area and number of tumor spheroids after IL-1β treatment. Similar results were observed in primary patient cells (Additional file 1: Figure S3). These data thus suggest that the expression of CEBPD is critical in glioma spheroid formation under IL-1β stimulation.

### The identification of CEBPD-regulated genes in U373MG cells

**Fig. 3:**
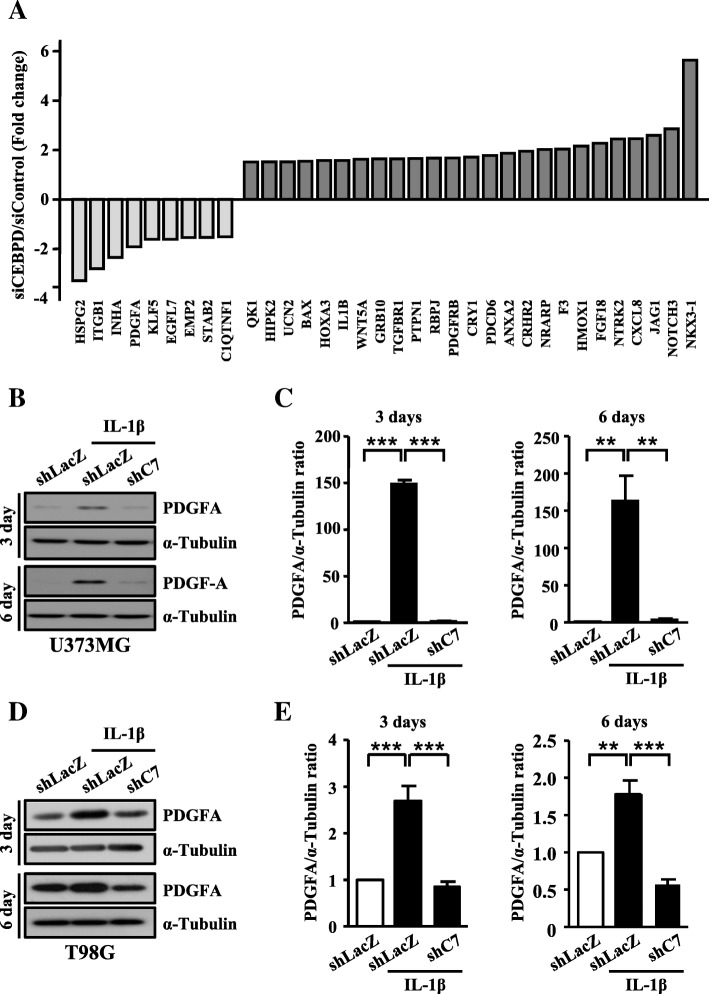
CEBPD correlates to PDGFA expression in U373MG and T98G spheroid cells. **a** Microarray analysis performed using total RNA harvested from U373MG cells with transiently knockdown of CEBPD by siRNA. In Gene Ontology databases, the differentially expressed genes of U373MG cells transfected with siRNA are listed. **b**–**e** CEBPD deficiency impairs PDGFA expression with IL-1β treatment in U373MG or T98G spheroid cells. Western blot analyses performed using protein lysates harvested from U373MG or T98G spheroid cells stably expressing shLacZ or shC7 treated with or without 5 ng/mL IL-1β on day 3 and 6. Expression of α-tubulin served as the internal control. Cells transduced with shLacZ served as the sham control. (Total data were obtained from three independent experiments, and the data shown here were from one representative assay. All data are expressed as mean ± SEM. Differences among groups were determined with one-way ANOVA followed by Tukey’s multiple comparison test. **p < 0.01, ***p < 0.001) shLacZ, shβ-galactosidase; shC7, shCEBPD

To date, little was known about the relationship between CEBPD-regulated genes and stemness of GSCs. To identify the critical genes regulated by CEBPD in glioma, genome-wide profiling and comparisons were conducted using U373MG cells with and without the siRNA-mediated knockdown of CEBPD. As shown in Additional file 1: Table S1, we found that the expression of 342 genes were significantly reduced, and 510 genes were significantly increased in U373MG cells with CEBPD knockdown. Among these CEBPD-responsive genes, PDGFA (platelet-derived growth factor subunit A), downregulated in CEBPD-knockdown U373MG cells (Fig. 3a), was of great interest in our study because of its critical role in tumor progression [21, 22].

### PDGFA gene is a direct target of CEBPD

**Fig. 4:**
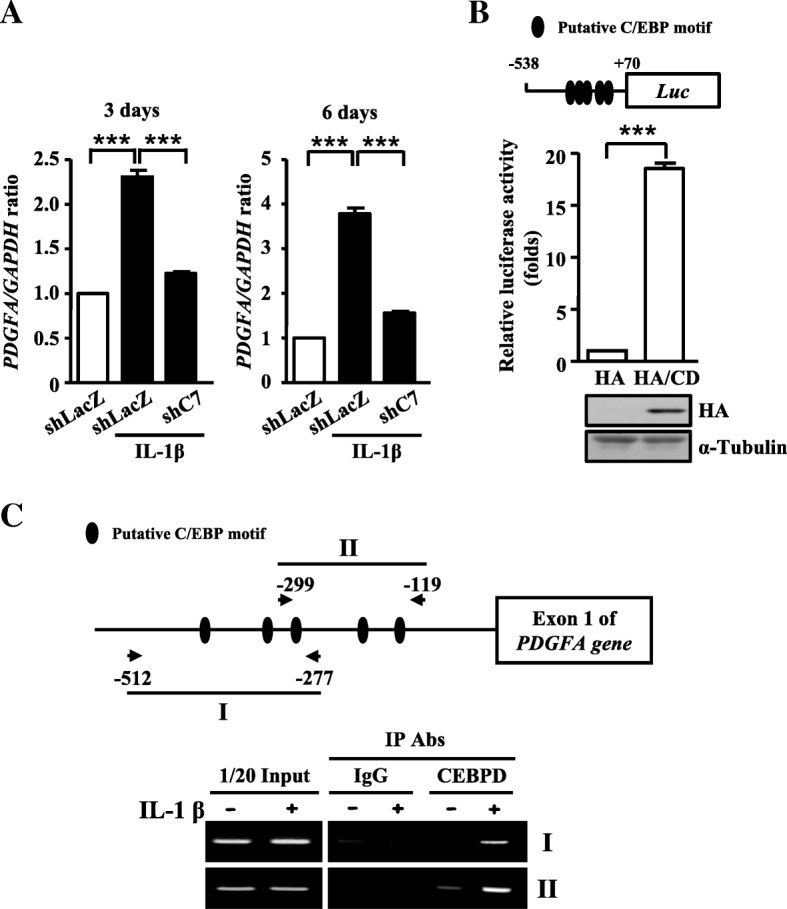
CEBPD regulates *PDGFA* transcription through *PDGFA* promoter region. **a** The *PDGFA* mRNA levels in U373MG spheroid cells stably expressing shLacZ or shC7 cells. Real-time PCR analyses conducted with specific primers using cDNA harvested from 5 ng/mL IL-1β treatment on day 3 and 6. Cells transduced with shLacZ served as the sham control. **b** The identification of CEBPD binding motifs on the *PDGFA* promoter region. The representation of reporter construct is at the upper region. The reporter vector carrying the *PDGFA* promoter was co-transfected with the indicated expression vectors in U373MG cells. After 12 h, the cell lysates were harvested for Western blot analysis and luciferase assay. Expression of α-tubulin served as the internal control. **c** CEBPD binding to the *PDGFA* promoter in vivo. Chromatin from U373MG spheroid cells treated with or without 5 ng/mL IL-1β was isolated, and chromatin immunoprecipitation assays were performed with indicated antibodies. The precipitated DNA was amplified using specific primers of the *PDGFA* promoter region. (Total data were obtained from three independent experiments, and the data shown here were from one representative assay. All data are expressed as mean ± SEM. Differences among groups were determined with one-way ANOVA followed by Tukey’s multiple comparison test (**a**) or unpaired two-tailed *t* test (**b**). ***p < 0.001) shLacZ, shβ-galactosidase; shC7, shCEBPD

Previous studies have shown that PDGFA, which binds and activates PDGF receptor tyrosine kinases, promotes cancer cell proliferation, invasion, and self-renewal [2, 23]. However, the role of PDGFA in glioma in the inflammatory environment remains to be elucidated. We found that IL-1β-induced increase of PDGFA protein expression was remarkably attenuated in CEBPD-knockdown U373MG and T98G spheroid cells (Fig. 3b–e). *PDGFA* mRNA expression also showed a similar result in U373MG spheroid cells with or without IL-1β treatment and CEBPD knockdown (Fig. 4a). Combined, these results suggest a strong link between PDGFA and CEBPD. Due to CEBPD’s function as a transcription factor, we then investigated if PDGFA was a downstream target of CEBPD. Using luciferase reporter assay, we identified a potent CEBPD-responsive region in *PDGFA* promoter at − 538/+ 70 bp (Fig. 4b). In addition, in vivo DNA binding assay showed that the binding of CEBPD on the *PDGFA* promoter was observed in the presence of IL-1β in U373MG spheroid cells (Fig. 4c). These data thus suggest that CEBPD regulates *PDGFA* transcription by binding to the *PDGFA* promoter region.

### Recombinant protein PDGFAA rescues glioma stem cell properties in CEBPD-lacking spheroid cells

**Fig. 5:**
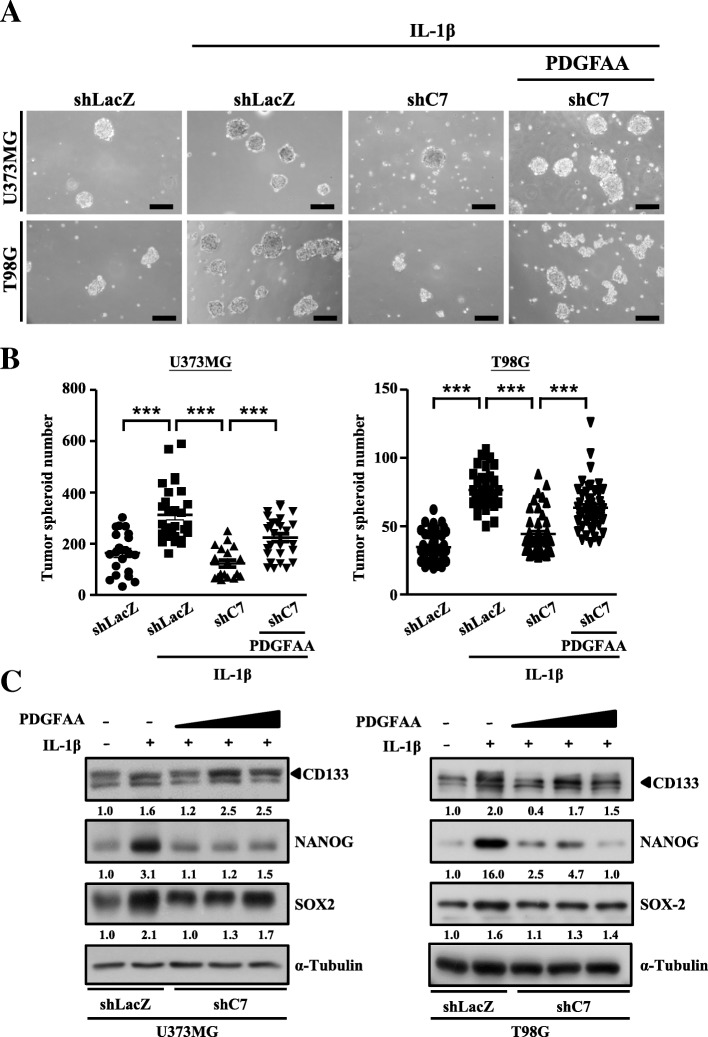
PDGFAA rescues the glioma stem-like cell feature in U373MG and T98G cells lacking CEBPD under IL-1β treatment. **a** Images collected from tumor spheroids of U373MG or T98G cells stably expressing shLacZ or shC7 treated with or without 5 ng/mL IL-1β or PDGFAA on day 6. Scale bar, 0.2 mm. **b** Quantitative analysis of tumor spheroid number in **a** using ImageJ software. **c** Western blot analyses conducted with antibodies as indicated using protein lysates from U373MG or T98G spheroid cells stably expressing shLacZ or shC7 treated with or without 5 ng/mL IL-1β or PDGFAA on day 6. Expression of α-tubulin served as the internal control. Cells transduced with shLacZ served as the sham control. (Total data were obtained from three independent experiments, and the data shown here were from one representative assay. All data are expressed as mean ± SEM. Differences among groups were determined with one-way ANOVA followed by Tukey’s multiple comparison test. ***p < 0.001) shLacZ, shβ-galactosidase; shC7, shCEBPD

Previous studies have demonstrated that PDGFA contributes to cancer stemness [2, 22], but the molecular mechanisms driving PDGFA expression in GSCs under IL-1β treatment are still not elucidated. To determine the role of PDGFA in CEBPD-mediated stemness of GSCs under IL-1β treatment, we tested whether the exogenous PDGFAA was able to reverse the inhibitory effects caused by CEBPD knockdown. The result showed that recombinant protein PDGFAA successfully rescued the self-renewal ability of U373MG and T98G (increase of spheroid formation) with knockdown of CEBPD (Fig. 5a, b). PDGFAA also partially reversed the effect of CEBPD knockdown-mediated downregulation of CD133, SOX2, and NANOG expressions (Fig. 5c). Combined, these observations suggest that CEBPD regulates the GSCs stemness through PDGFA under inflammatory stimulation.

### PDGFA expression is associated with poorer prognosis in GBM patients

**Fig. 6:**
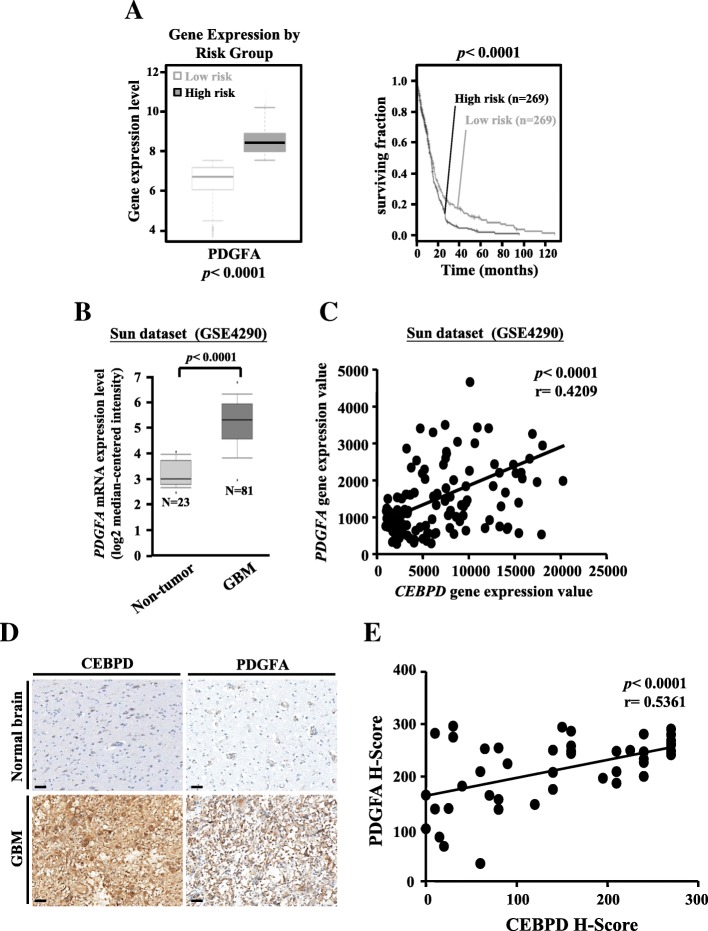
PDGFA expression is clinically relevant in glioma patients. **a** High-risk glioma correlated with the increased PDGFA expression, and the Kaplan-Meier survival curves of TCGA GBM patients indicated the correlation between low expressions of PDGFA in patients and the better overall survival significantly compared to patients with high PDGFA expressions. **b** Analyses of the Sun dataset (GSE4290) showing a higher expression of PDGFA in GBM than in NSC or non-tumor samples. **c** *PDGFA* gene expression value correlated with *CEBPD* expression in glioma samples from the Sun dataset (GSE4290). **d** Representative immunohistochemistry analyses of CEBPD and PDGFA in GBM and normal brain tissues. Scale bar, 50 μm. **e** PDGFA *H*-score value correlated with CEBPD *H*-score in glioma samples. (Data are expressed as mean ± SEM. Differences between groups were determined with the unpaired two-tailed *t* test for **a** and **b**. Pearson’s correlation (*r*) values are indicated for **c** and **f**)

We next examined whether PDGFA expression was associated with the histopathology of glioma and patient prognosis. The overall survival of patients with low *PDGFA* expression exhibited better prognosis than those with high *PDGFA* expression (Fig. 6a). Consistently, glioma samples expressed higher *PDGFA* than non-tumor brain tissue (Fig. 6b). Similar results have been observed in the Bredel and Lee databases (Additional file 1: Figure S4a). We also found that the expression level of *PDGFA* significantly correlated with the expression level of *CEBPD* in these glioma samples (Fig. 6c and Additional file 1: Figure S4b). Furthermore, we examined the protein expression of PDGFA and CEBPD in tissue arrays of human glioma samples by immunohistochemical analyses. We found that the expression of PDGFA was significantly higher in glioma samples and was correlated with the CEBPD expression levels in these glioma samples (Fig. 6d, e and Additional file 1: Table S2). Taken together, these data suggest that the expression of PDGFA is increased in glioma and is correlated with CEBPD expression.

## Discussion

The tumor microenvironment influences cancer initiation, promotion, and malignancy [1, 3, 24]. Approximately 30–50% of the cells in gliomas are microglia or macrophages, and the tumor-associated macrophages/microglia (TAM) plays crucial roles in tumor growth, invasion, and metastasis [3]. TAM can secrete inflammatory factors and promote cancer stem cell formation. However, the mechanism of how inflammation induces GSC spheroid formation still remains largely unclear. Our data demonstrate a novel function of CEBPD, which promotes glioma stem cell spheroid formation under the inflammatory environment. We show that CEBPD is responsive to IL-1β stimulation and enhances the transcription and expression of *PDGFA*, which, in turn, promotes glioma stemness. IL-1β, IL-6, TGF-β, and EGF have been shown to promote glioma invasion from tumor-associated macrophage secretion [3]. Our previous studies also show that these factors can also induce CEBPD expression [9, 10, 17]. However, whether inflammatory cytokines promote stemness is still unclear. Herein, we provide evidences showing the upregulation of PDGFA expression and promotion of glioma stemness by CEBPD under inflammatory stimulation.

CEBPD plays a functional role in tumor formation and progression. Low expression of CEBPD in a variety of cancers has been observed, including hepatocellular carcinoma and cervical cancer, and the hypermethylation in the CEBPD promoter mediated by polycomb group complex and DNA methyltransferase contributes to the silencing of CEBPD expression at this context [15, 25]. The overexpression of CEBPD in these cancers can induce cancer cell apoptosis and inhibit cell growth, thus implying CEBPD’s role as a tumor suppressor. On the other hand, recent studies have demonstrated that CEBPD plays an oncogenic role when endogenous CEBPD expression is relatively higher or during inducible CEBPD expression in some cancer types, such as bladder cancer and lung cancer [17, 26]. In these cancer cells, CEBPD can promote drug resistance, invasion, and lymphangiogenesis [17, 26]. However, the function of CEBPD in glioma stem cell spheroid formation remains less well understood. The GEO dataset shows that CEBPD is highly expressed in GBM compared to normal brain tissue. Additionally, we demonstrate that IL-1β can induce CEBPD transcription and expression in glioma cells, which, in turn, promotes glioma stemness. Our observations thus favor the model of CEBPD function promoting tumor growth in glioma.

A previous study suggests that constitutively active STAT3 confers cellular transformation, survival, proliferation, anti-apoptosis, and chemosensitivity in several human malignancies [17]. Moreover, STAT3 is known for its functions in regulating stem cell transcription factors and to maintain the stem cell phenotype and growth and proliferation of GSCs [2, 27]. In a bladder urothelial carcinoma model, CEBPD expression is upregulated by STAT3 activation and promotes cancer drug resistance [17]. It is thus possible that STAT3 not only regulates the stemness transcription factors directly, but also indirectly regulates the stemness transcription factors through CEBPD expression. On the other hand, STAT3 activation can be stimulated through inflammatory factors and cytokines, such as IL-6 and EGF [17, 28]. Studies also show that IL-1β can activate STAT3 in osteoblasts, Th17, and glioma [29–31]. According to bioinformatic datasets and previous studies, the glioma-associated microenvironment can increase expression levels of inflammatory factors and cytokines [1, 3]. We thus propose a model of signaling pathways in the STAT3/CEBPD/PDGFA axis mediating inflammatory factor-induced stemness in glioblastoma. Further investigation of STAT3 function in glioblastoma could thus provide a better understanding of the detailed mechanism of inflammatory factor-induced stemness at this context.

PDGFA signaling maintains glioma stem cell growth and self-renewal [2, 23]. The stimulation of PDGFA signaling has been observed in GBMs and is associated with tumor initiation and malignant progression. Furthermore, PDGFA signaling can also increase STAT3 activation through autocrine or paracrine PDGFAA stimulation [2, 32]. PDGF can form various homo- or heterodimers, such as PDGFA/A, PDGFB/B, PDGFA/B, PDGFC/C, and PDGFD/D [21, 22]. Activation of two structurally related cell membrane tyrosine kinase receptors (PDGFR-A and PDGFR-B) can affect various physiological and pathological functions through PDGF binding [21, 22]. Our data show that CEBPD affects PDGFRA expression through CEBPD knockdown array profile (transient and stable CEBPD-knockdown U373MG cells). In the future, we will continue investigating how CEBPD regulates PDGFRA expression in glioma.

Interestingly, we show that *IL-1β* is upregulated when CEBPD expression is decreased in the siRNA experiment on U373 cells (Fig. 3a). Our previous works show that CEBPD responsive miRNAs, including miR-4300 and miR-135a, are increased in CEBPD-overexpressing U373MG cells [33]. According to microRNA target prediction softwares (Targetscan, miRWalk, and miRanda), miR-4300 and miR-135a can target to *IL-1β* 3′UTR and may further attenuate *IL-1β* expression. On the other hand, CEBPD knockdown has been found to enhance the expression of several IL-1β-induced inflammatory genes including interleukin-8, intracellular adhesion molecule-1, monocyte chemoattractant protein-1, and IL-1β in human brain pericytes [34]. IL-1β is a potent inflammatory stimulus, and its induction following CEBPD knockdown would appear to contradict its anti-inflammatory roles in astrocytes. Whether these changes induced by CEBPD knockdown actually lead to altered inflammatory events in vivo is still unclear, and further studies investigating the underlying mechanisms controlling this response are required.

## Conclusions

In this study, we have demonstrated that IL-1β-induced CEBPD promotes glioma stemness in U373MG, T98G, and PT#3-spheroid cells by elevating PDGFA expression. In addition, our data validate the suppression of CEBPD by knockdown system to inhibit the stemness effects through inhibition of PDGFA-induced stem cell markers (Additional file 1: Figure S5). This finding thus highlights the importance of CEBPD and PDGFA regulation and implies both as potential targets for novel therapeutic strategies against malignant glioma in inflammatory environments.

## Additional file

Additional file 1: **Table S1.** CEBPD-responsive genes in U373MG cells. **Table S2.** *H*-score data shown each of the patient IHC images. **Figure S1.** CEBPD contributes to self-renewal in U373MG and T98G spheroid cells under IL-1β treatment. **Figure S2.** Transient knockdown of CEBPD in U373MG cells reduces tumor sphere formation and attenuates stem cell transcription factor expression. **Figure S3.** Transient knockdown of CEBPD in PT#3 cells reduces tumor spheroid formation and attenuates stem cell transcription factor expression. **Figure S4.** PDGFA expression is clinically relevant in GBM patients. **Figure S5.** A schematic diagram illustrating the proposed model for the modulatory effect of CEBPD on glioma stem-like cell formation after IL-1β treatment. (PPTX 6917 kb)

## Data Availability

The datasets generated and analyzed during the current study are available from the corresponding authors on reasonable request.

## Abbreviations

CEBPD: CCAAT/enhancer-binding protein delta
GBM: Glioblastoma multiforme
GSCs: Glioma stem-like cells
IL-1β: Interleukin-1 beta
PDGFA: Platelet-derived growth factor subunit A
TAM: Tumor-associated macrophages/microglia
TNF-α: Tumor necrosis factor-alpha
