# Supplementary material for: CCAAT/enhancer-binding protein delta regulates the stemness of glioma stem-like cells through activating PDGFA expression upon inflammatory stimulation
Source: J Neuroinflammation. 2019 Jul 12;16:146. doi: 10.1186/s12974-019-1535-z (PMC6626372; doi:10.1186/s12974-019-1535-z)
Supplement: Supplementary file 1 — Table S1. CEBPD-responsive genes in U373MG cells. Table S2. H-score data shown each of the patient IHC images. Figure S1. CEBPD contributes to self-renewal in U373MG and T98G spheroid cells under IL-1β treatment. Figure S2. Transient knockdown of CEBPD in U373MG cells reduces tumor sphere formation and attenuates stem cell transcription factor expression. Figure S3. Transient knockdown of CEBPD in PT#3 cells reduces tumor spheroid formation and attenuates stem cell transcription factor expression. Figure S4. PDGFA expression is clinically relevant in GBM patients. Figure S5. A schematic diagram illustrating the proposed model for the modulatory effect of CEBPD on glioma stem-like cell formation after IL-1β treatment. (PPTX 6917 kb) [file 12974_2019_1535_MOESM1_ESM.pptx]

## Slide 1
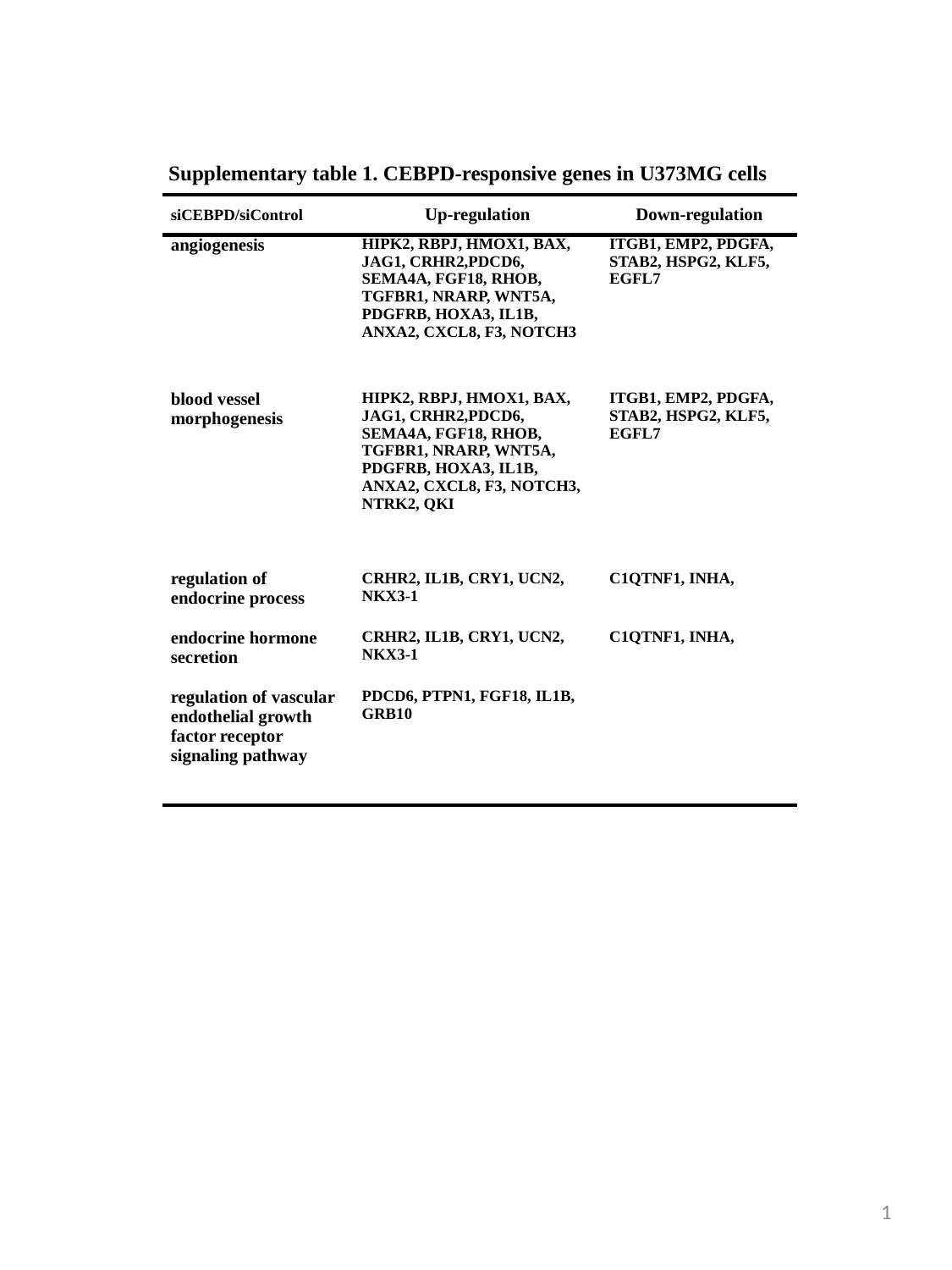

Supplementary table 1. CEBPD-responsive genes in U373MG cells
| siCEBPD/siControl | Up-regulation | Down-regulation |
| --- | --- | --- |
| angiogenesis | HIPK2, RBPJ, HMOX1, BAX, JAG1, CRHR2,PDCD6, SEMA4A, FGF18, RHOB, TGFBR1, NRARP, WNT5A, PDGFRB, HOXA3, IL1B, ANXA2, CXCL8, F3, NOTCH3 | ITGB1, EMP2, PDGFA, STAB2, HSPG2, KLF5, EGFL7 |
| blood vessel morphogenesis | HIPK2, RBPJ, HMOX1, BAX, JAG1, CRHR2,PDCD6, SEMA4A, FGF18, RHOB, TGFBR1, NRARP, WNT5A, PDGFRB, HOXA3, IL1B, ANXA2, CXCL8, F3, NOTCH3, NTRK2, QKI | ITGB1, EMP2, PDGFA, STAB2, HSPG2, KLF5, EGFL7 |
| regulation of endocrine process | CRHR2, IL1B, CRY1, UCN2, NKX3-1 | C1QTNF1, INHA, |
| endocrine hormone secretion | CRHR2, IL1B, CRY1, UCN2, NKX3-1 | C1QTNF1, INHA, |
| regulation of vascular endothelial growth factor receptor signaling pathway | PDCD6, PTPN1, FGF18, IL1B, GRB10 | |
1

## Slide 2
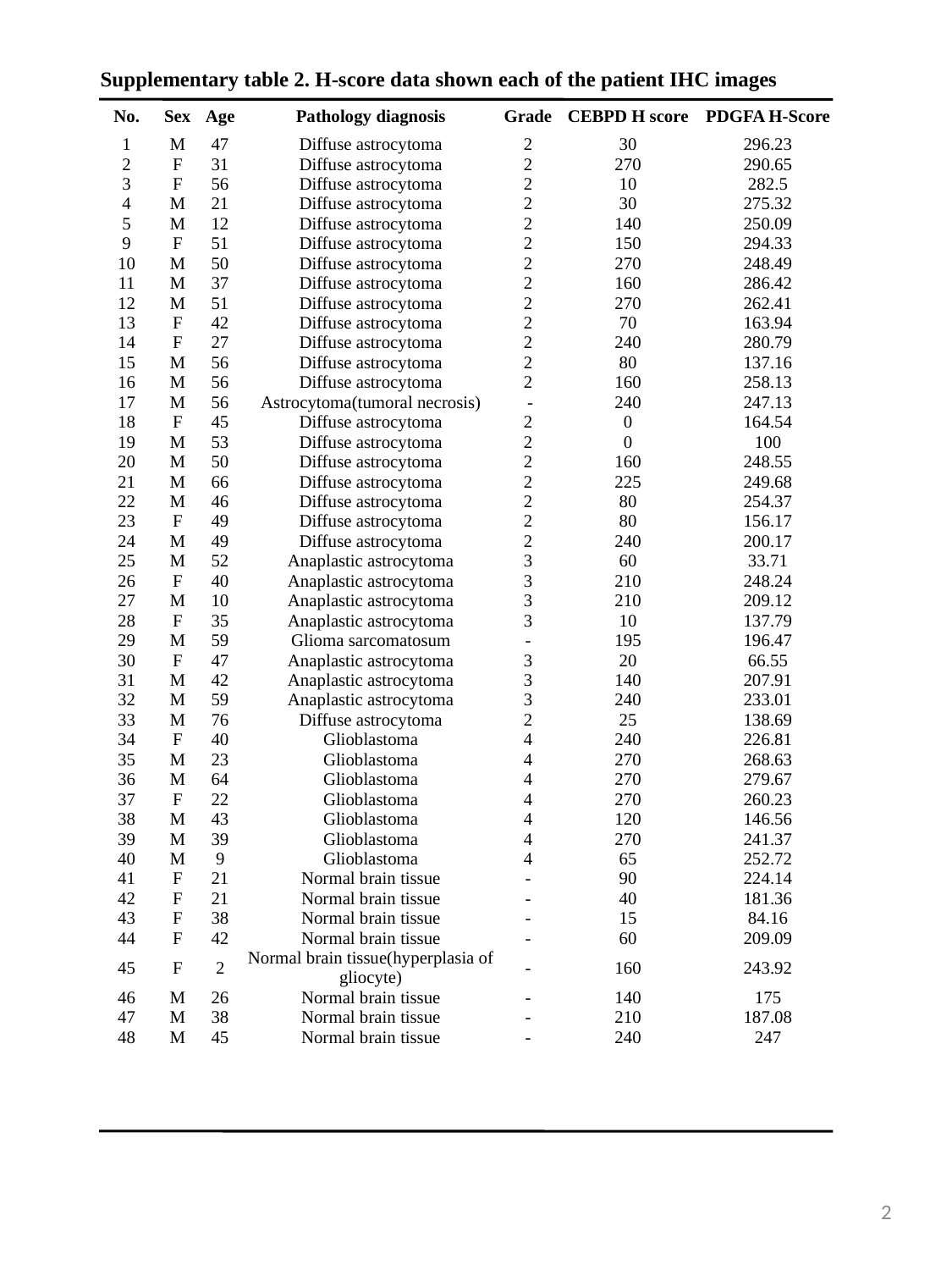

| Supplementary table 2. H-score data shown each of the patient IHC images | | | | | | |
| --- | --- | --- | --- | --- | --- | --- |
| No. | Sex | Age | Pathology diagnosis | Grade | CEBPD H score | PDGFA H-Score |
| 1 | M | 47 | Diffuse astrocytoma | 2 | 30 | 296.23 |
| 2 | F | 31 | Diffuse astrocytoma | 2 | 270 | 290.65 |
| 3 | F | 56 | Diffuse astrocytoma | 2 | 10 | 282.5 |
| 4 | M | 21 | Diffuse astrocytoma | 2 | 30 | 275.32 |
| 5 | M | 12 | Diffuse astrocytoma | 2 | 140 | 250.09 |
| 9 | F | 51 | Diffuse astrocytoma | 2 | 150 | 294.33 |
| 10 | M | 50 | Diffuse astrocytoma | 2 | 270 | 248.49 |
| 11 | M | 37 | Diffuse astrocytoma | 2 | 160 | 286.42 |
| 12 | M | 51 | Diffuse astrocytoma | 2 | 270 | 262.41 |
| 13 | F | 42 | Diffuse astrocytoma | 2 | 70 | 163.94 |
| 14 | F | 27 | Diffuse astrocytoma | 2 | 240 | 280.79 |
| 15 | M | 56 | Diffuse astrocytoma | 2 | 80 | 137.16 |
| 16 | M | 56 | Diffuse astrocytoma | 2 | 160 | 258.13 |
| 17 | M | 56 | Astrocytoma(tumoral necrosis) | - | 240 | 247.13 |
| 18 | F | 45 | Diffuse astrocytoma | 2 | 0 | 164.54 |
| 19 | M | 53 | Diffuse astrocytoma | 2 | 0 | 100 |
| 20 | M | 50 | Diffuse astrocytoma | 2 | 160 | 248.55 |
| 21 | M | 66 | Diffuse astrocytoma | 2 | 225 | 249.68 |
| 22 | M | 46 | Diffuse astrocytoma | 2 | 80 | 254.37 |
| 23 | F | 49 | Diffuse astrocytoma | 2 | 80 | 156.17 |
| 24 | M | 49 | Diffuse astrocytoma | 2 | 240 | 200.17 |
| 25 | M | 52 | Anaplastic astrocytoma | 3 | 60 | 33.71 |
| 26 | F | 40 | Anaplastic astrocytoma | 3 | 210 | 248.24 |
| 27 | M | 10 | Anaplastic astrocytoma | 3 | 210 | 209.12 |
| 28 | F | 35 | Anaplastic astrocytoma | 3 | 10 | 137.79 |
| 29 | M | 59 | Glioma sarcomatosum | - | 195 | 196.47 |
| 30 | F | 47 | Anaplastic astrocytoma | 3 | 20 | 66.55 |
| 31 | M | 42 | Anaplastic astrocytoma | 3 | 140 | 207.91 |
| 32 | M | 59 | Anaplastic astrocytoma | 3 | 240 | 233.01 |
| 33 | M | 76 | Diffuse astrocytoma | 2 | 25 | 138.69 |
| 34 | F | 40 | Glioblastoma | 4 | 240 | 226.81 |
| 35 | M | 23 | Glioblastoma | 4 | 270 | 268.63 |
| 36 | M | 64 | Glioblastoma | 4 | 270 | 279.67 |
| 37 | F | 22 | Glioblastoma | 4 | 270 | 260.23 |
| 38 | M | 43 | Glioblastoma | 4 | 120 | 146.56 |
| 39 | M | 39 | Glioblastoma | 4 | 270 | 241.37 |
| 40 | M | 9 | Glioblastoma | 4 | 65 | 252.72 |
| 41 | F | 21 | Normal brain tissue | - | 90 | 224.14 |
| 42 | F | 21 | Normal brain tissue | - | 40 | 181.36 |
| 43 | F | 38 | Normal brain tissue | - | 15 | 84.16 |
| 44 | F | 42 | Normal brain tissue | - | 60 | 209.09 |
| 45 | F | 2 | Normal brain tissue(hyperplasia of gliocyte) | - | 160 | 243.92 |
| 46 | M | 26 | Normal brain tissue | - | 140 | 175 |
| 47 | M | 38 | Normal brain tissue | - | 210 | 187.08 |
| 48 | M | 45 | Normal brain tissue | - | 240 | 247 |
2

## Slide 3
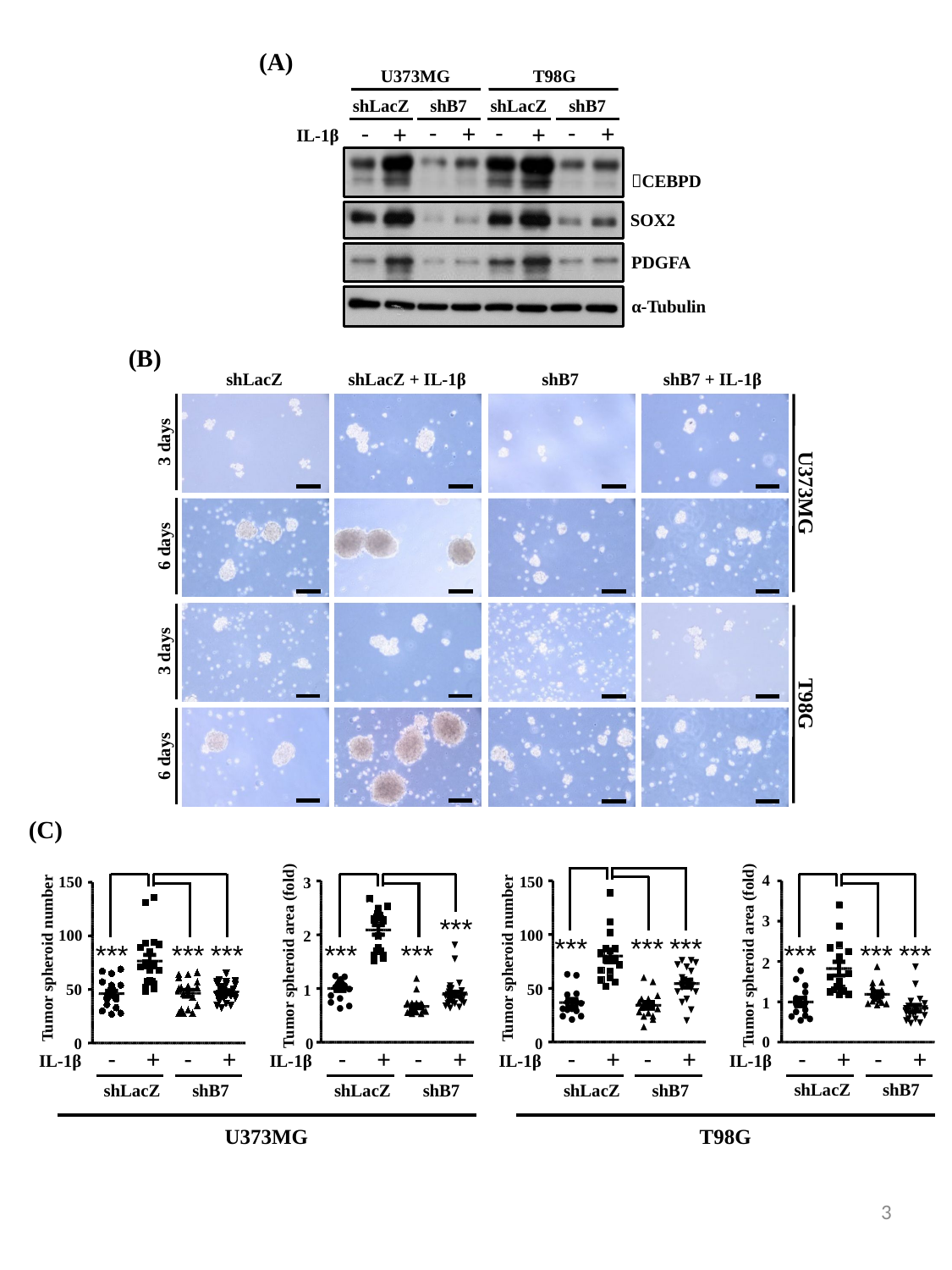

(A)
U373MG
T98G
shLacZ
shB7
shLacZ
shB7
-
-
-
-
+
+
+
+
IL-1β
CEBPD
SOX2
PDGFA
α-Tubulin
(B)
shLacZ
shLacZ + IL-1β
shB7
shB7 + IL-1β
3 days
U373MG
6 days
3 days
T98G
6 days
(C)
***
***
***
150
100
***
***
***
Tumor spheroid number
50
0
4
150
3
***
***
***
***
***
***
3
100
2
Tumor spheroid area (fold)
Tumor spheroid area (fold)
Tumor spheroid number
2
50
1
1
0
0
0
-
-
+
+
IL-1β
shB7
shLacZ
-
-
+
+
IL-1β
shB7
shLacZ
-
-
+
+
IL-1β
shB7
shLacZ
-
-
+
+
IL-1β
shB7
shLacZ
U373MG
T98G
3

## Slide 4
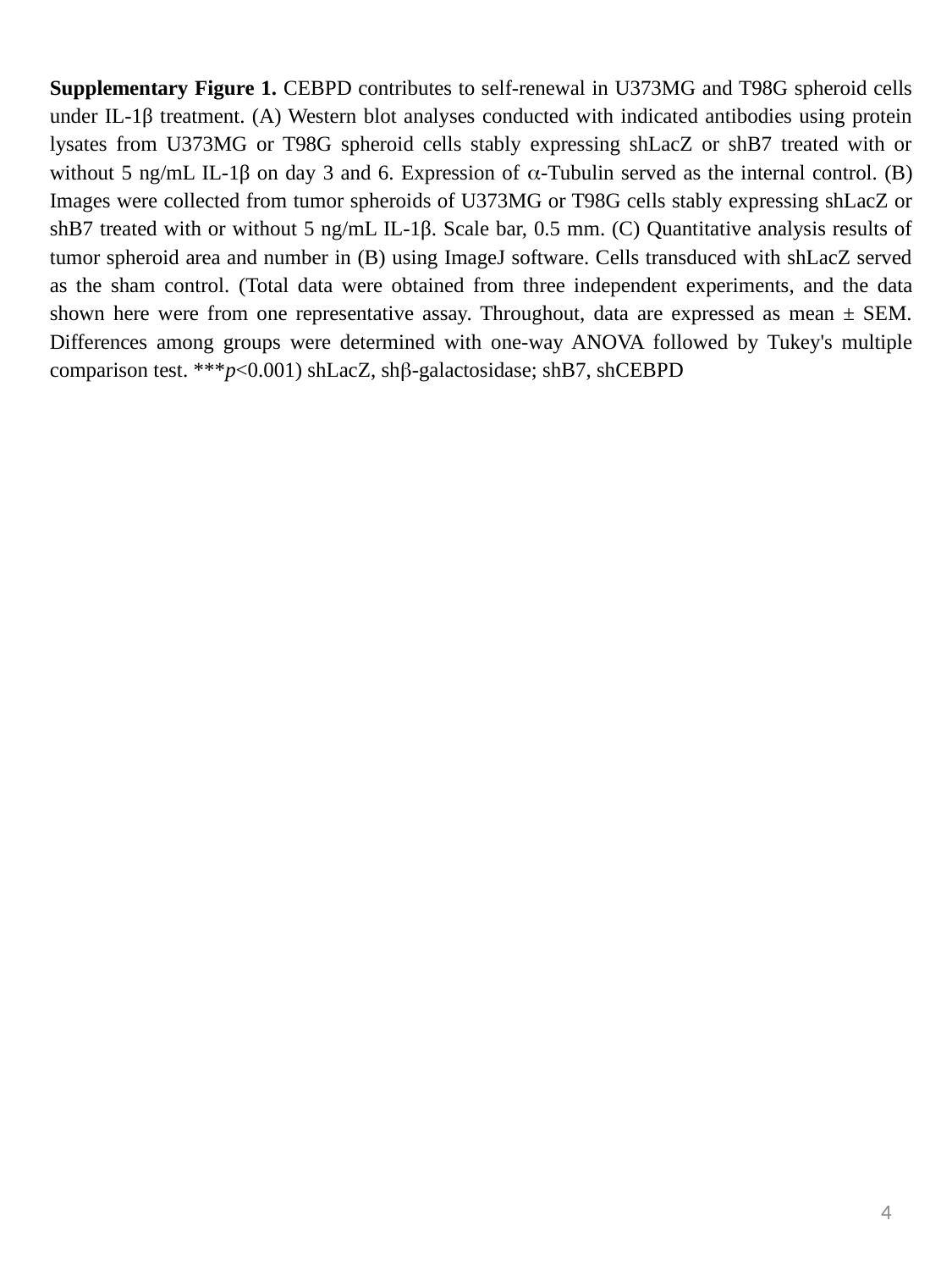

Supplementary Figure 1. CEBPD contributes to self-renewal in U373MG and T98G spheroid cells under IL-1β treatment. (A) Western blot analyses conducted with indicated antibodies using protein lysates from U373MG or T98G spheroid cells stably expressing shLacZ or shB7 treated with or without 5 ng/mL IL-1β on day 3 and 6. Expression of a-Tubulin served as the internal control. (B) Images were collected from tumor spheroids of U373MG or T98G cells stably expressing shLacZ or shB7 treated with or without 5 ng/mL IL-1β. Scale bar, 0.5 mm. (C) Quantitative analysis results of tumor spheroid area and number in (B) using ImageJ software. Cells transduced with shLacZ served as the sham control. (Total data were obtained from three independent experiments, and the data shown here were from one representative assay. Throughout, data are expressed as mean ± SEM. Differences among groups were determined with one-way ANOVA followed by Tukey's multiple comparison test. ***p<0.001) shLacZ, shb-galactosidase; shB7, shCEBPD
4

## Slide 5
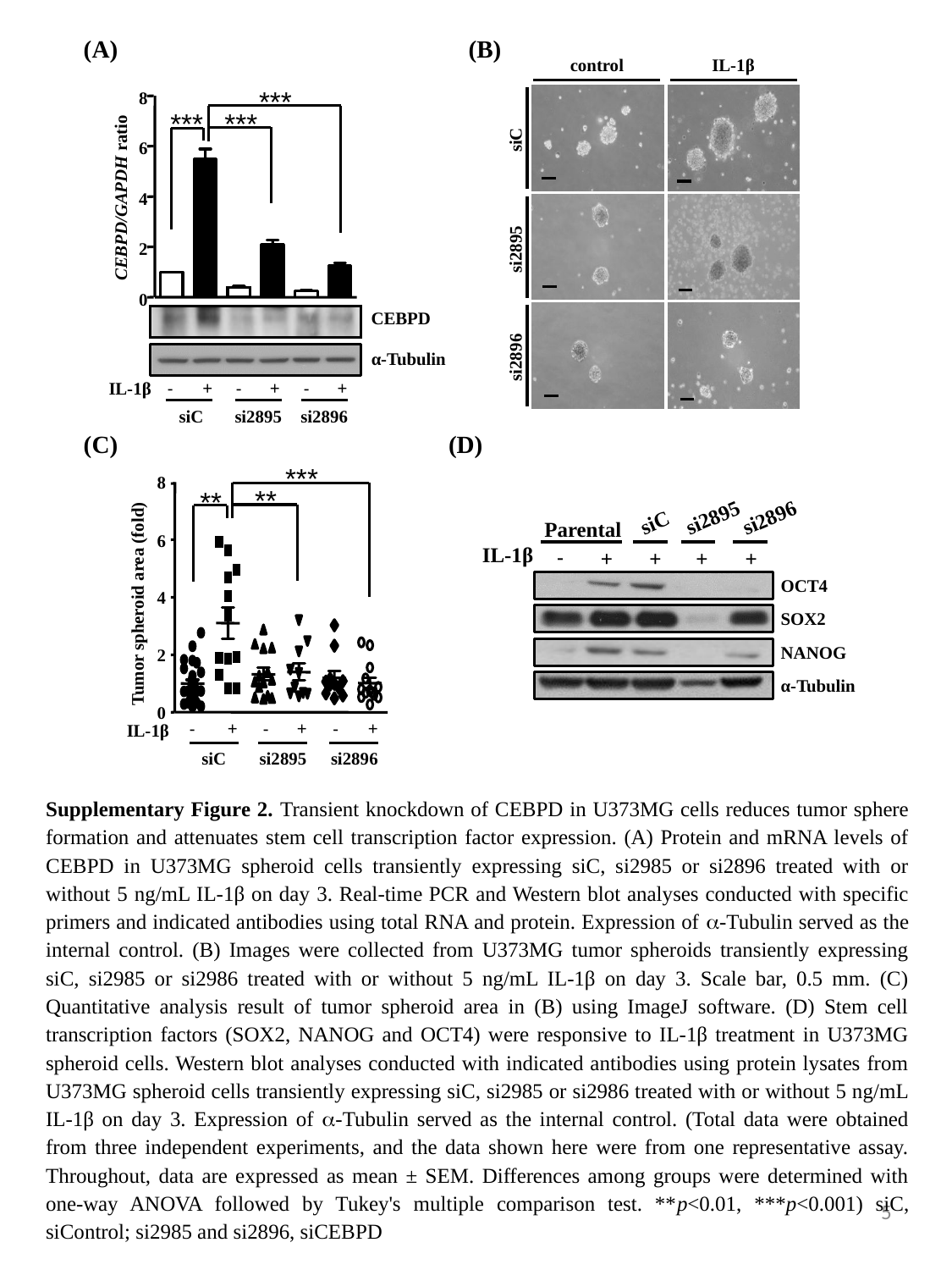

(A)
(B)
control
IL-1β
siC
si2895
si2896
***
***
***
8
6
CEBPD/GAPDH ratio
4
2
0
CEBPD
α-Tubulin
IL-1β
-
+
-
+
-
+
siC
si2895
si2896
(C)
(D)
***
**
**
8
si2895
siC
si2896
Parental
IL-1β
-
+
+
+
+
OCT4
SOX2
NANOG
α-Tubulin
6
4
Tumor spheroid area (fold)
2
0
-
+
-
+
-
+
IL-1β
siC
si2895
si2896
Supplementary Figure 2. Transient knockdown of CEBPD in U373MG cells reduces tumor sphere formation and attenuates stem cell transcription factor expression. (A) Protein and mRNA levels of CEBPD in U373MG spheroid cells transiently expressing siC, si2985 or si2896 treated with or without 5 ng/mL IL-1β on day 3. Real-time PCR and Western blot analyses conducted with specific primers and indicated antibodies using total RNA and protein. Expression of a-Tubulin served as the internal control. (B) Images were collected from U373MG tumor spheroids transiently expressing siC, si2985 or si2986 treated with or without 5 ng/mL IL-1β on day 3. Scale bar, 0.5 mm. (C) Quantitative analysis result of tumor spheroid area in (B) using ImageJ software. (D) Stem cell transcription factors (SOX2, NANOG and OCT4) were responsive to IL-1β treatment in U373MG spheroid cells. Western blot analyses conducted with indicated antibodies using protein lysates from U373MG spheroid cells transiently expressing siC, si2985 or si2986 treated with or without 5 ng/mL IL-1β on day 3. Expression of a-Tubulin served as the internal control. (Total data were obtained from three independent experiments, and the data shown here were from one representative assay. Throughout, data are expressed as mean ± SEM. Differences among groups were determined with one-way ANOVA followed by Tukey's multiple comparison test. **p<0.01, ***p<0.001) siC, siControl; si2985 and si2896, siCEBPD
5

## Slide 6
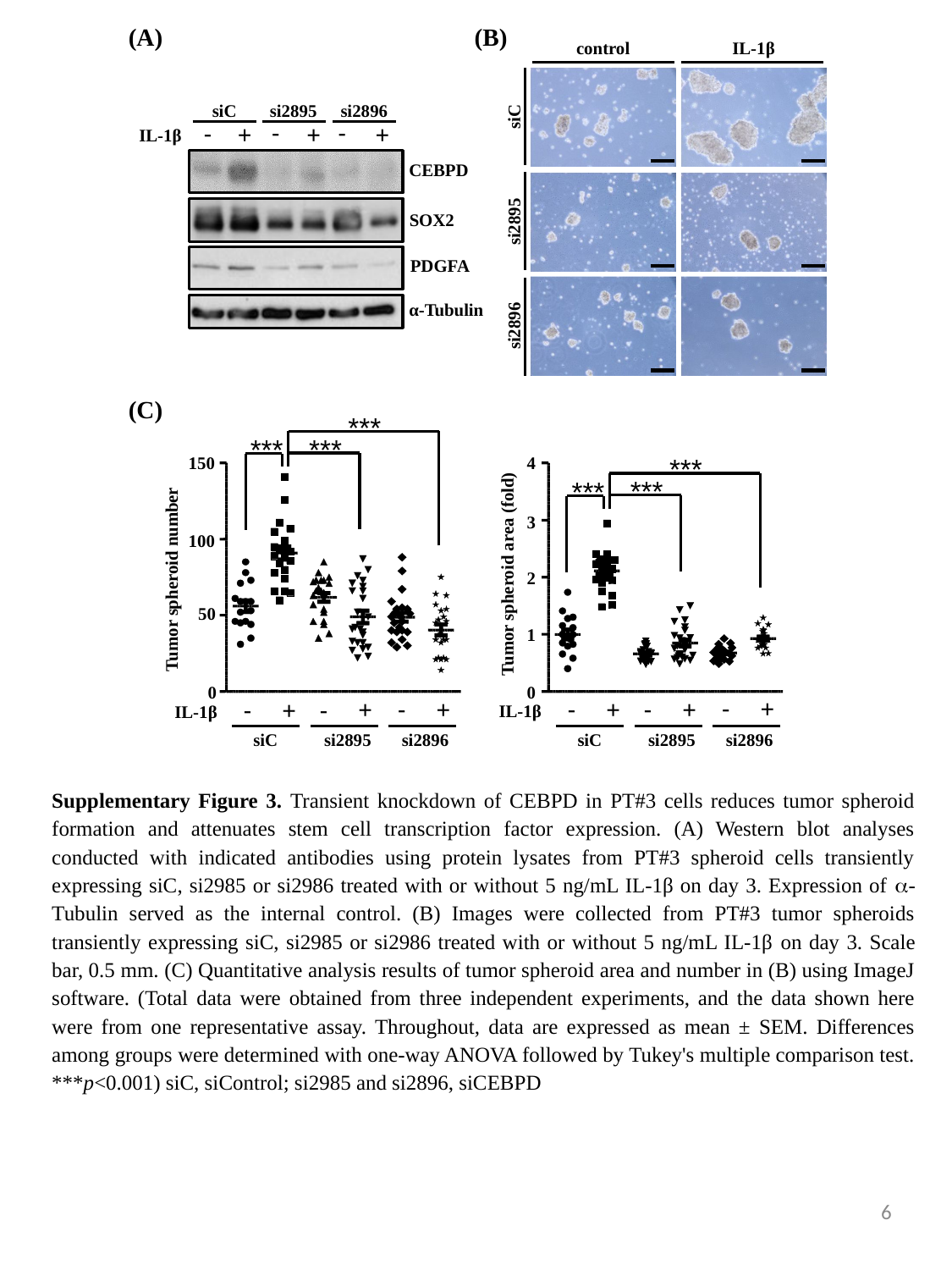

(A)
(B)
control
IL-1β
siC
si2895
si2896
siC
si2895
si2896
-
-
-
+
+
+
IL-1β
CEBPD
SOX2
PDGFA
α-Tubulin
(C)
***
***
***
***
***
***
150
4
3
100
Tumor spheroid area (fold)
2
Tumor spheroid number
50
1
0
0
-
-
-
-
+
-
-
+
+
+
+
+
IL-1β
IL-1β
siC
si2895
si2896
siC
si2895
si2896
Supplementary Figure 3. Transient knockdown of CEBPD in PT#3 cells reduces tumor spheroid formation and attenuates stem cell transcription factor expression. (A) Western blot analyses conducted with indicated antibodies using protein lysates from PT#3 spheroid cells transiently expressing siC, si2985 or si2986 treated with or without 5 ng/mL IL-1β on day 3. Expression of a-Tubulin served as the internal control. (B) Images were collected from PT#3 tumor spheroids transiently expressing siC, si2985 or si2986 treated with or without 5 ng/mL IL-1β on day 3. Scale bar, 0.5 mm. (C) Quantitative analysis results of tumor spheroid area and number in (B) using ImageJ software. (Total data were obtained from three independent experiments, and the data shown here were from one representative assay. Throughout, data are expressed as mean ± SEM. Differences among groups were determined with one-way ANOVA followed by Tukey's multiple comparison test. ***p<0.001) siC, siControl; si2985 and si2896, siCEBPD
6

## Slide 7
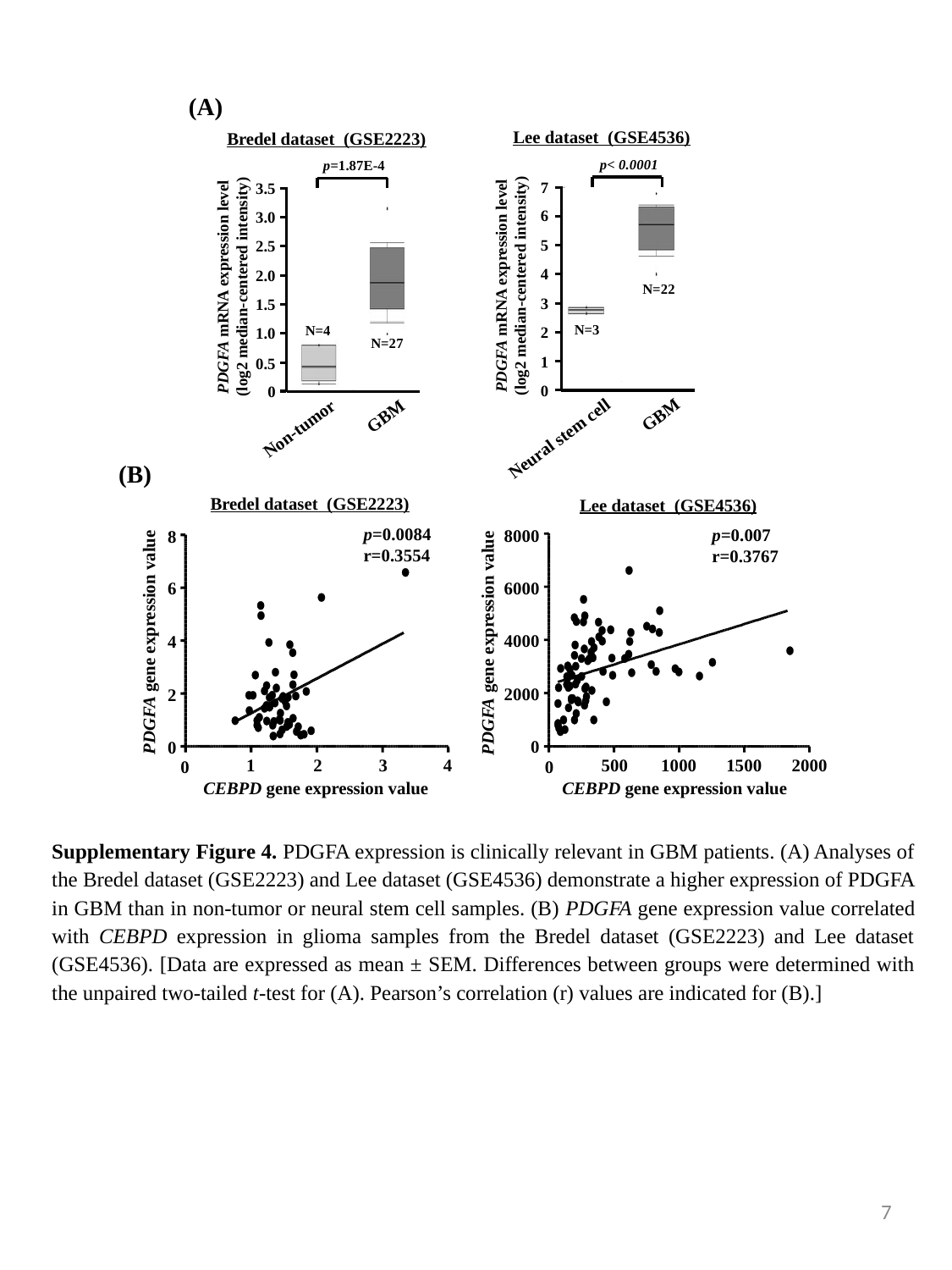

(A)
Lee dataset (GSE4536)
p< 0.0001
7
6
5
PDGFA mRNA expression level
(log2 median-centered intensity)
4
N=22
3
N=3
2
1
0
GBM
Neural stem cell
Bredel dataset (GSE2223)
p=1.87E-4
3.5
3.0
2.5
PDGFA mRNA expression level
(log2 median-centered intensity)
2.0
1.5
N=4
1.0
N=27
0.5
0
GBM
Non-tumor
(B)
Bredel dataset (GSE2223)
p=0.0084
r=0.3554
8
6
PDGFA gene expression value
4
2
0
1
2
3
4
0
CEBPD gene expression value
Lee dataset (GSE4536)
p=0.007
r=0.3767
8000
6000
PDGFA gene expression value
4000
2000
0
500
1000
1500
2000
0
CEBPD gene expression value
Supplementary Figure 4. PDGFA expression is clinically relevant in GBM patients. (A) Analyses of the Bredel dataset (GSE2223) and Lee dataset (GSE4536) demonstrate a higher expression of PDGFA in GBM than in non-tumor or neural stem cell samples. (B) PDGFA gene expression value correlated with CEBPD expression in glioma samples from the Bredel dataset (GSE2223) and Lee dataset (GSE4536). [Data are expressed as mean ± SEM. Differences between groups were determined with the unpaired two-tailed t-test for (A). Pearson’s correlation (r) values are indicated for (B).]
7

## Slide 8
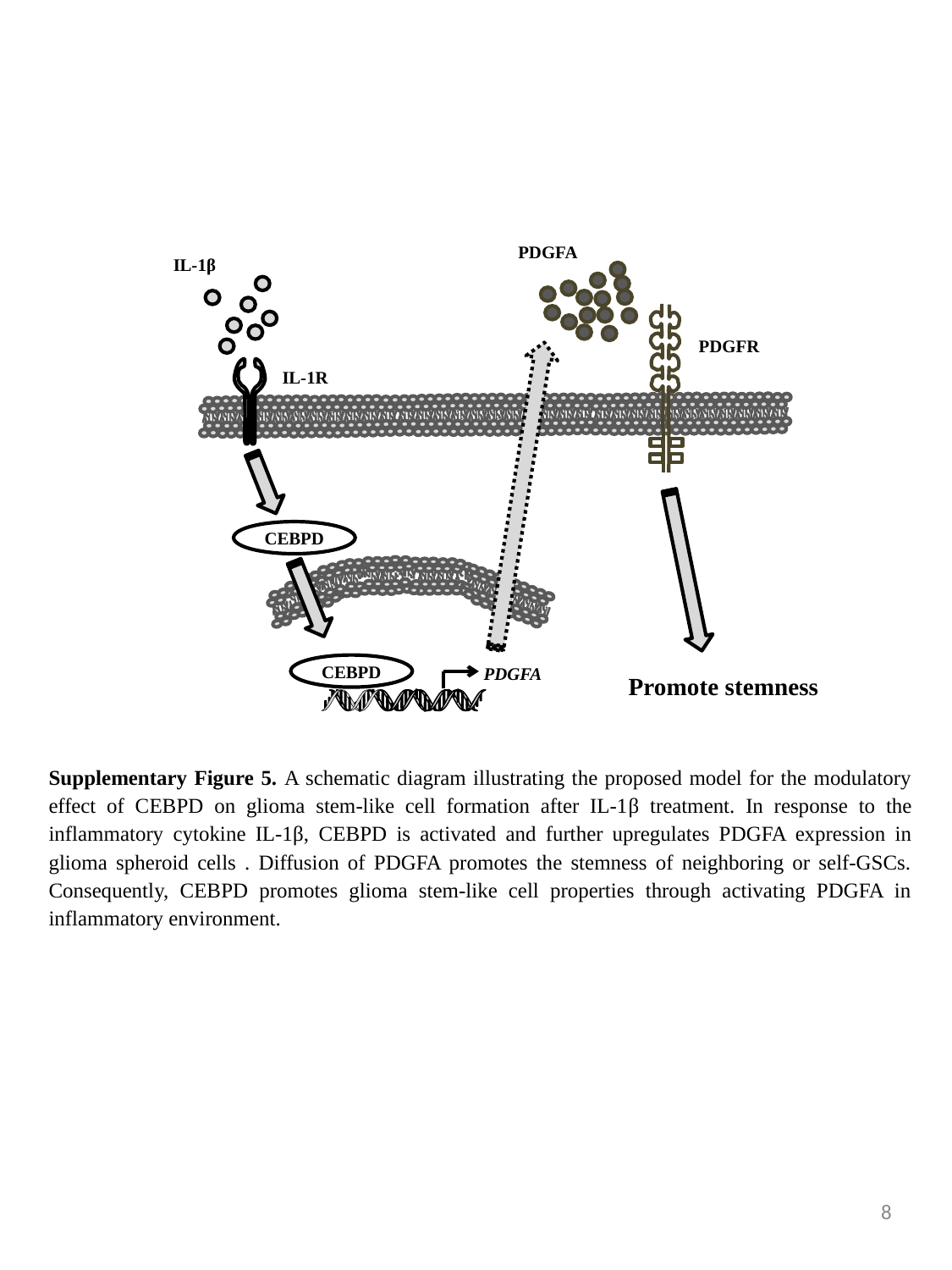

PDGFA
IL-1β
PDGFR
IL-1R
CEBPD
CEBPD
PDGFA
Promote stemness
Supplementary Figure 5. A schematic diagram illustrating the proposed model for the modulatory effect of CEBPD on glioma stem-like cell formation after IL-1β treatment. In response to the inflammatory cytokine IL-1β, CEBPD is activated and further upregulates PDGFA expression in glioma spheroid cells . Diffusion of PDGFA promotes the stemness of neighboring or self-GSCs. Consequently, CEBPD promotes glioma stem-like cell properties through activating PDGFA in inflammatory environment.
8
